# Association of American Dentists

**Published:** 1856-09

**Authors:** 


*Association of American Dentists.*—This body assembled in New York, on Wednesday, Aug. 6th, John R. Rich, Esq., President, in the chair. Mr. Chevalier, of New York, manufacturer of dental instruments, was admitted a member, by a vote of 17 to 14. Mr. Kearsing, of Brooklyn, manufacturer of dental apparatus, was refused admission. The following officers were chosen:

*President*, Dr. Chapin A. Harris, of Baltimore.
*Vice-President*, Dr. Daniel Neal, of Philadelphia.
*Recording Secretary*, Dr. Elisha Townsend, of Philadelphia.
*Corresponding Secretary*, Dr. W. W. Allport, of Chicago.

---

*Statistics.*—We notice in the Cincinnati Medical Observer, that in Ohio, physicians are required to make returns of the births and deaths coming under their observation. For this purpose they are to be furnished with blanks, by the Secretary of State. In this respect Ohio is ahead Massachusetts, and her vital and mortuary statistics will undoubtedly prove of great accuracy and value. When shall we have a law obliging physicians, instead of undertakers, to make mortuary returns?—*Boston Med. & Surg. Journal.*

